# Bio-based 3-hydroxypropionic- and acrylic acid production from biodiesel glycerol via integrated microbial and chemical catalysis

**DOI:** 10.1186/s12934-015-0388-0

**Published:** 2015-12-21

**Authors:** Tarek Dishisha, Sang-Hyun Pyo, Rajni Hatti-Kaul

**Affiliations:** Biotechnology, Center for Chemistry and Chemical Engineering, Lund University, 221 00 Lund, Sweden; Department of Microbiology and Immunology, Faculty of Pharmacy, Beni-Suef University, 62511 Beni-Suef, Egypt

**Keywords:** Biocatalysis, Cascade enzymatic reaction, *Lactobacillus reuteri*, *Gluconobacter oxydans*, Catalytic dehydration, Titanium dioxide, 3-Hydroxypropionic acid, Acrylic acid, 1,3-Propanediol, Biodiesel glycerol

## Abstract

**Background:**

3-Hydroxypropionic acid (3HP) and acrylic acid (AA) are industrially important platform- and secondary chemical, respectively. Their production from renewable resources by environment-friendly processes is desirable. In the present study, both chemicals were almost quantitatively produced from biodiesel-derived glycerol by an integrated process involving microbial and chemical catalysis.

**Results:**

Glycerol was initially converted in a fed-batch mode of operation to equimolar quantities of 3HP and 1,3-propanediol (1,3PDO) under anaerobic conditions using resting cells of *Lactobacillus reuteri* as a biocatalyst. The feeding rate of glycerol was controlled at 62.5 mg/g_CDW_.h which is half the maximum metabolic flux of glycerol to 3HP and 1,3PDO through the *L. reuteri* propanediol-utilization (*pdu*) pathway to prevent accumulation of the inhibitory intermediate, 3-hydroxypronionaldehyde (3HPA). Subsequently, the cell-free supernatant containing the mixture of 3HP and 1,3PDO was subjected to selective oxidation under aerobic conditions using resting cells of *Gluconobacter oxydans* where 1,3PDO was quantitatively converted to 3HP in a batch system. The optimum conditions for the bioconversion were 10 g/L substrate and 5.2 g/L cell dry weight. Higher substrate concentrations led to enzyme inhibition and incomplete conversion. The resulting solution of 3HP was dehydrated to AA over titanium dioxide (TiO_2_) at 230 °C with a yield of >95 %.

**Conclusions:**

The present study represents the first report on an integrated process for production of acrylic acid at high purity and -yield from glycerol through 3HP as intermediate without any purification step. The proposed process could have potential for industrial production of 3HP and AA after further optimization.

**Graphical abstract:**
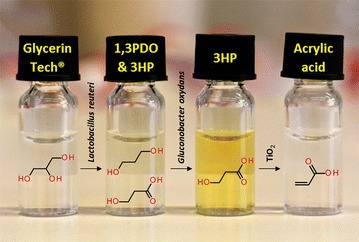
Integrated three-step process for conversion of biodiesel glycerol to 3-hydroxypropionic acid (3HP) and acrylic acid (AA). Glycerol was initially converted to equimolar quantities of 3HP and 1,3-propanediol (1,3PDO) using resting cells of *Lactobacillus reuteri*. Subsequently, the cell-free supernatant containing the mixture of 3HP and 1,3PDO was subjected to selective oxidation using resting cells of *Gluconobacter oxydans* where 1,3PDO was quantitatively converted to 3HP. The resulting solution of 3HP was dehydrated to AA over titanium dioxide (TiO2) at 230 °C.

**Electronic supplementary material:**

The online version of this article (doi:10.1186/s12934-015-0388-0) contains supplementary material, which is available to authorized users.

## Background

Significant efforts are continuously being made around the world to move from the current fossil-based economy to a more sustainable economy based on renewable resources. In order to match the efficiency and flexibility of the petrochemical industry, the bio-based industry needs to develop a set of versatile building blocks, or platforms from which a range of products can be derived [1, 2]. Taking this into consideration, the Department of Energy, USA has identified 30 platform chemicals composed of 1–6 carbon atoms as potential candidates for bio-based production [2]. Polyols and organic acids constitute the majority of these chemicals; examples are glycerol, propionic acid and 3-hydroxypropionic acid (3HP) [1, 2].

Acrylic acid (AA) is a bulk chemical with annual production capacity of 4.2 million metric tons [3]. The major application of acrylate is its use in water-based acrylic resins in the form of acrylate esters [4], which are used in decorative, masonry and industrial coatings and also in adhesives, binders for paper, leather and textile, polishes, carpet backing compounds and tablet coatings [5, 6]. Glacial AA is also used for the manufacture of super absorbent polymers used in disposable diapers [4]. The current industrial production of AA involves gas-phase catalytic oxidation of fossil-based propylene via acrolein as intermediate [4, 6]. Besides the non-renewable raw material, acrolein is highly toxic and explosive. The process is estimated to result in emissions of 3.25 kg CO_2_ eq per kg AA, shared equally between the raw materials plus the process, and waste treatment [7]. Most of the alternative chemical production routes were abandoned as they were found to be economically unattractive, use non-environmental friendly catalysts or have low acrylate yields [6].

Many renewable routes for production of AA using bio-based raw materials have been reported such as direct fermentation of lactate using *Clostridium propionicum* [8], catalytic dehydration of lactic acid or 3HP obtained by fermentation [9–12], and catalytic conversion of microbially-produced fumaric acid [13]. Although lactic acid is less toxic and is produced industrially via fermentation at a concentration exceeding 10 %, production of acrylate by fermentation of lactate is limited by a strong product inhibition [8]. Additionally, catalytic dehydration of the secondary hydroxyl group of lactic acid has proven to be difficult, because it is quite resistant toward hydrolysis [11, 12]. On the other hand, β-hydroxy acid (3HP) was shown to be more easily dehydrated into AA than the α-hydroxy acid (lactic acid) [9, 10, 14]. This was the main trigger for several companies and research groups to develop novel renewable, economically feasible routes for production of 3HP as a potential source for AA [14].

3HP is a structural isomer of lactic acid (2-hydroxypropionic acid). Its two functionalities, hydroxyl- and carboxyl groups, make it a versatile compound for organic synthesis [15–17]. It can also be incorporated as a cross-linking agent in coatings, lubricants and antistatic agents for textiles [18]. Other applications are expected in food industry, cosmetics and fertilizers [19].

Industrial production of 3HP has not yet been established due to lack of economic feasibility and/or environmental compatibility for most of the suggested chemical production routes [15, 18]. Nevertheless, the high expectation for 3HP as a platform chemical gives a projected market volume of 3.6 million ton/year [3].

Biologically, 3HP can be obtained in small quantities as an end product of glycerol and acrylate metabolism by few wild-type microorganisms [18]. Jiang et al. and Henry et al. have suggested different metabolic pathways for the production of 3HP from glucose and glycerol [20, 21], and many have been practically evaluated [22–26]. However, most of these biological production routes are limited by low 3HP yield, and the need for nitrogen source/cofactors for maintaining the production process as well as for product recovery and purification thereafter which increase the production cost [22–26].

The rapid expansion of the biofuels market, especially biodiesel and bioethanol, has resulted in accumulation of surplus amounts of glycerol as a by-product. In order to convert glycerol to 3HP, two different pathways have been described; all share the first step of selective dehydration of glycerol yielding 3-hydroxypropionaldehyde (3HPA) in a reaction catalyzed by glycerol/diol dehydratase. The resulting aldehyde is then oxidized forming 3HP through aldehyde dehydrogenase, or through the three-step cascade reaction catalyzed by propionaldehyde dehydrogenase (PduP), phosphotransacylase (PduL), and kinase (PduW) [20]. However, for achieving the redox balance other products are produced simultaneously such as 1,3-propanediol (1,3PDO) through 1,3-propanediol oxidoreductase (PduQ).

In an earlier study, the biocatalytic dehydration of glycerol and dismutation of the resulting 3HPA was achieved yielding equimolar amounts of 1,3PDO and 3HP using resting cells of *Lactobacillus reuteri*, hence resulting in 50 mol % yield of 3HP from glycerol [27]. *L. reuteri* is an obligate heterofermentative lactic acid bacterium that does not utilize glycerol as a carbon source but instead as an indirect electron acceptor [28, 29]. The metabolism of glycerol takes place through propanediol-utilization (Pdu) pathway comprising five enzymes, vitamin B12-dependent glycerol/diol dehydratase (PduCDE) catalyzing the dehydration of glycerol to 3HPA, PduQ for reduction of 3HPA to 1,3 PDO, and PduP, PduL and PduW catalyzing oxidation of 3HPA to 3HP. 3HPA flux through these pathways is influenced by the level of NADH inside the microbial cell; higher NADH levels resulting in reduction of 3HPA (normal case with growing cells in which the reduced cofactor is generated as a result of sugar metabolism), while accumulation of NAD^+^ favors the oxidative pathway (the case with resting cells).

Acetic acid bacteria possess several alcohol dehydrogenases capable of selective oxidation of alcohols to the corresponding aldehydes and ketones. The resulting aldehydes can be further oxidized to the corresponding carboxylic acids by the action of aldehyde dehydrogenases working in cascade with the alcohol dehydrogenase [30]. Acetic acid bacteria are used industrially as catalysts for the oxidation of ethanol to acetic acid, glucose to glucuronic acid, glycerol to dihydroxyacetone, and many other processes [30–35]. *Gluconobacter oxydans* was earlier used as a biocatalyst for selective oxidation of 2-methyl-1,3-propanediol to 2-methyl-3-hydroxypropionic acid [31], a reaction not easily achievable by chemical catalysis.

**Fig. 1:**
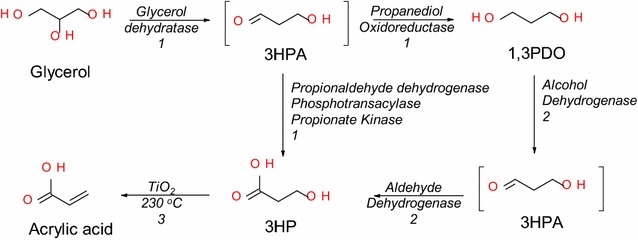
Schematic representation of the three-step process for conversion of glycerol to AA through 3HP. The proposed three-step process for the conversion of glycerol to 3-hydroxypropionic acid and acrylic acid. Step numbers are indicated on the *arrows*. *1* Reactions catalyzed by *L. reuteri*, *2* Reactions catalyzed by *G. oxydans* and *3* Reaction catalyzed by TiO_2_

The present study demonstrates the production of AA from biodiesel glycerol via integrated three-step process. Glycerol was initially converted to 1,3 PDO and 3HP using resting cells of *L. reuteri* under anaerobic conditions. The product mixture was then subjected to selective biological oxidation under aerobic conditions using resting cells of *G. oxydans* resulting in 3HP. The resultant acid was catalytically dehydrated using TiO_2_ to AA (Fig. 1).

## Results and discussion

### Fed-batch biotransformation of glycerol to 1,3PDO and 3HP using resting cells of *L. reuteri*

Using *L. reuteri* cells as biocatalyst for transformation of glycerol requires induction of expression of the genes encoding the enzymes and structural proteins in the Pdu pathway, by including small amounts of glycerol or 1,2-propanediol in the cultivation medium with a sugar carbon source [36]. Co-metabolism of glycerol with the main carbon source results in higher cell density, as regeneration of NAD^+^ resulting from the conversion of 3HPA to 1,3PDO diverts the sugar metabolism to acetate (via pyruvate and acetyl-phosphate) yielding ATP instead of being reduced to ethanol and lactate [37, 38].

Our earlier study on the analysis of glycerol fluxes towards 3HPA, 1,3PDO and 3HP revealed that the rate of formation of 3HPA is higher than the rate for its further conversion to 3HP and 1,3PDO [27]. The maximum specific production rates of 3HP and 1,3PDO using wild-type *L. reuteri* at pH 5 were 62.4 and 52.7 mg/g_CellDryWeight_ h, respectively [27]. Considering these maxima, the maximum specific glycerol consumption rate yielding only 1,3PDO and 3HP without accumulation of the intermediate 3HPA was calculated to be 128.3 mg_Gly_/g_CDW_ h by summing up the molar specific production rates for 1,3PDO and 3HP using the following equation:

$$q_{Gly} = q_{3HP} + q_{1,3PDO}$$

**Fig. 2:**
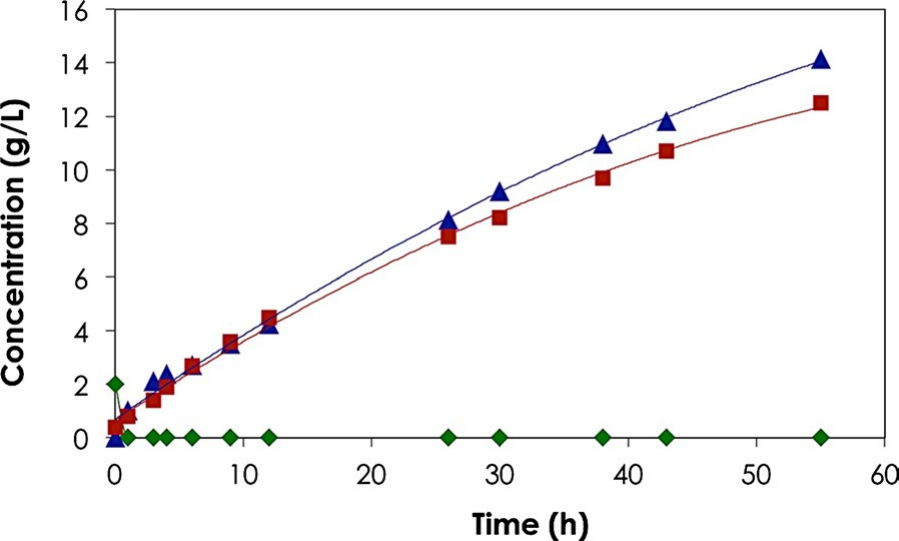
Biotransformation of glycerol to 3HP and 1,3PDO. Fed-batch biotransformation of glycerol using resting cells of *L. reuteri* (12 g_CDW_/L). Glycerol (100 g/L) was fed at a rate of 0.75 g_gly_/h. Symbols: concentrations of glycerol (*filled diamond*), 3HP (*filled triangle*) and 1,3PDO (*filled square*)

Since 12 g dry weight of biocatalyst is used in the present study, the specific consumption rate was 1.54 g_Gly_/12 g_CDW_ h. Using a feeding rate of 0.75 g_Gly_/h (half of the maximum rate), conversion of 40 g glycerol and production of high amounts of 3HP (19.9 g) and 1,3PDO (17.5 g) at a rate of 0.35 and 0.3 g/h, respectively, were achieved. The increase in the reaction volume caused by the feeding solution resulted in final concentration of 14 and 12 g/L for 3HP and 1,3PDO, respectively (Fig. 2). The molar ratio of 1,3PDO to 3HP was 1 mol/mol ensuring the balanced production for maintaining redox equivalence, and their corresponding yields were 0.42 g_1,3PDO_/g_Gly_ and 0.48 g_3HP_/g_Gly_. No accumulation of the intermediate aldehyde and/or product inhibition was observed during the whole biotransformation process.

The co-production of 1,3PDO and 3HP is mediated by a co-factor (NADH-NAD^+^) recycling between the oxidative and reductive branches of the metabolic pathway which is essential for continuity of the biotransformation reaction. Hence, directing the reaction towards 3HP as a single product by knocking out the gene encoding 1,3-propanediol oxidoreductase seems unfeasible. One realistic approach is to oxidize the produced 1,3PDO to 3HP in a separate step.

### Selective oxidation of 1,3PDO to 3HP using resting cells of *G. oxydans*

**Fig. 3:**
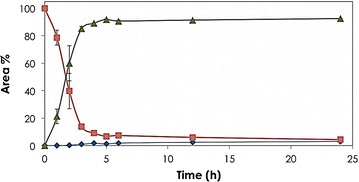
Biotransformation of 1,3PDO to 3HP. Time course for biotransformation of 1,3PDO (10 mg/mL) using resting cells of *G. oxydans* (5.2 mg_CDW_/mL). The figure shows the % peak area of 1,3PDO (*filled square*), 3HPA (*filled diamond*) and 3HP (*filled diamond*). Biotransformation (1 mL working volume) was done in 5 mL vial placed in a thermomixer at 28 °C and 800 rpm

Bioconversion of 1,3PDO to 3HP by the resting cells of *G. oxydans* was first investigated by varying the biocatalyst concentration (2.6, 3.9, 5.2 and 6.5 mg_CDW_/mL using 10 mg/mL substrate) and 1,3PDO concentration (5, 10, 15, 20, 25 and 30 mg/mL using 5.2 mg_CDW_/mL biocatalyst), the results of which are summarized in Table 1. The reaction was studied under uncontrolled pH in batch mode of operation at 28 °C and 800 rpm in 1 mL working volume containing *G. oxydans* cells in aqueous substrate solution. Highest conversion of 91 % was achieved with 5 mg/mL 1,3PDO in 2 h reaction time. Over 90 % of 10 mg/mL 1,3PDO was converted in 5 h yielding 3HP as the main product with minute amounts of the intermediate 3HPA (Fig. 3). After 5 h, the conversion was marginal and was 96.9 % after 24 h.

**Table 1:** Biotransformation of 1,3PDO by *G. oxydans* at 28 °C and 800 rpm for 3 h

Biocatalyst (mg/mL)	Alcohol dehydrogenase reaction (ADH)			Aldehyde dehydrogenase reaction (ALDH)				3HP Yield ^d^ mol% (24 h)
	1,3PDO (mg/mL)	Remaining 1,3PDO (mg/mL)	Conversion^a^ (%)	Substrate^b^ (mg/mL)	3HPA (mg/mL)	3HP (mg/mL)	Conversion^c^ (%)	
5.2	5	0.2	95.3	4.6	0.2	5.4	95.0	95.4
5.2	10	1.4	86.1	8.4	0.1	10.1	98.9	96.9
5.2	15	7.7	48.7	7.1	0.1	8.6	99.1	97.1
5.2	20	13.7	31.3	6.1	0.1	7.3	98.6	96.6
5.2	25	18.9	24.5	6.0	1.4	5.5	76.0	98.5
5.2	30	24.8	17.2	5.0	1.6	4.2	68.0	98.3
2.6	10	4.5	54.9	5.4	0.0	6.5	99.7	98.5^e^
3.9	10	2.4	75.7	7.4	0.1	8.9	99.0	97.8^e^
6.5	10	2.0	80.2	7.8	0.2	9.3	98.1	95.8^e^

*1,3PDO* 1,3-propanediol, *3HPA* 3-hydroxypropionaldehyde, *3HP* 3-hydroxypropionic acid

^a^Conversion of 1,3PDO

^b^Substrate calculated as the sum of 3HPA and 3HP used in the ALDH reaction (converted to 3HPA equivalent)

^c^Conversion of 3HPA to 3HP

^d^Overall yield (mol%) of 3HP from 1,3PDO after 24 h

^e^calculated after 12 h

Higher substrate concentrations, 15–30 mg/mL resulted in 77.96, 66.71, 57.56 and 53.26 % conversion, respectively in 24 h. Slight accumulation of the intermediate 3HPA was observed at 25–30 mg/mL 1,3PDO. Two main enzymes are functional in this step including alcohol dehydrogenase (ADH) and an aldehyde dehydrogenase (ALDH) [30–35]. According to Wei et al. (2003) and our modeling/docking studies of 1,2-propanediol and 1,3PDO in the active site (data not shown), ethanol dehydrogenase is thought to be the enzyme responsible for oxidation of the propanediols to the corresponding aldehydes [32]. For the second step of oxidation of the aldehyde to the carboxylic acid, different aldehyde dehydrogenases were reported to be responsible, one is membrane-bound and the others are either membrane-bound or cytoplasmic enzyme(s). However, further studies are required to identify all the enzymes involved [32].

**Fig. 4:**
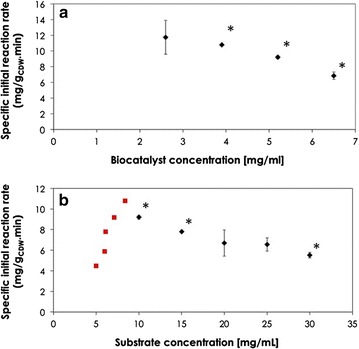
Effect of biocatalyst and 1,3PDO concentrations on specific initial oxidation rate by *G. oxydnas.* **a** Effect of biocatalyst concentration (mg/mL) on specific initial 1,3PDO (10 mg/mL) conversion rate (µg/mg_CDW_.min) using resting cells of *G. oxydans*. **b** Specific initial reaction rates of ADH (*filled diamond*) and ALDH (*filled square*) calculated from the data collected after 3 h during biotransformation of 1,3PDO using resting cells of *G. oxydans*. *Asterisk* indicates a significant difference between the marked data points. The data for the first substrate concentration (5 mg/ml) was ignored from Fig. 3b since the reaction was marginal after 2 h

The effects of biocatalyst concentration [mg_CDW_/mL] and substrate concentration [mg/mL] on specific initial reaction rate [mg/g_CDW_.min] are shown in Fig. 4. Although a higher biocatalyst concentration resulted in faster conversion rate, the specific initial reaction rate was significantly reduced (Fig. 4a). This could be a result of oxygen limitation that might occur at higher biomass concentration. On the other hand, the initial volumetric reaction rate was increased from 30.4 ± 5.3 µg/mL min using 2.6 mg_CDW_/mL to a maximum of 47.9 ± 0.8 µg/mL min using 5.2 mg_CDW_/mL. Increasing the cell concentration to 6.5 mg_CDW_/mL did not result in a significant increase in volumetric reaction rate. This explains the use of 5.2 mg_CDW_/mL for studying the effect of substrate concentration on biotransformation kinetics.

As seen in Fig. 4b, the rate of ADH catalyzed reaction decreased significantly with increase in substrate concentration from 10 to 30 mg/mL, suggesting substrate inhibition. Product inhibition could be ignored since the intermediate 3HPA was continuously converted to the corresponding acid and did not accumulate to an inhibitory level. On the other hand, increasing the substrate concentration for the ALDH catalyzed reaction was accompanied with increase in the reaction rate within the tested range. At initial 1,3PDO concentration of 25 and 30 mg/mL, accumulation of the intermediate aldehyde to 1.4 and 1.6 mg/mL, respectively, indicated that the overall rate of 1,3PDO oxidation to 3HPA catalyzed by ADH is higher than that for further oxidation of 3HPA to 3HP by the second enzyme in the cascade, ALDH.

**Fig. 5:**
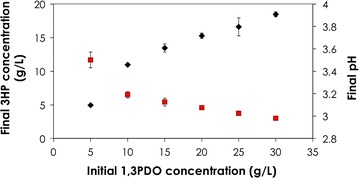
Effect of initial 1,3PDO concentration on final pH and 3HP concentration. Effect of initial 1,3PDO concentration (g/L) on 3HP concentration (g/L) (*filled diamond*) as well as final pH of the reaction (*filled square*) after 24 h. Biocatalyst concentration was 5.2 mg_CDW_/mL, reaction volume 1 mL in 5 mL vials, temperature was maintained at 28 °C and mixing at 800 rpm

The highest 3HP concentration obtained was 18.4 ± 0.4 mg/mL from 30 mg/mL 1,3PDO within 24 h. The final pH of the biotransformation reaction dropped from 3.5 ± 0.07 in case of 5 mg/mL 1,3PDO to 2.98 ± 0.01 with 30 mg/mL of 1,3PDO (Fig. 5). This sharp drop in the pH of the reaction might explain the marginal biotransformation of 1,3PDO observed after 5 h of the reaction since the optimal pH range for the alcohol and aldehyde dehydrogenases is between pH 5 and 7 [31–33].

**Fig. 6:**
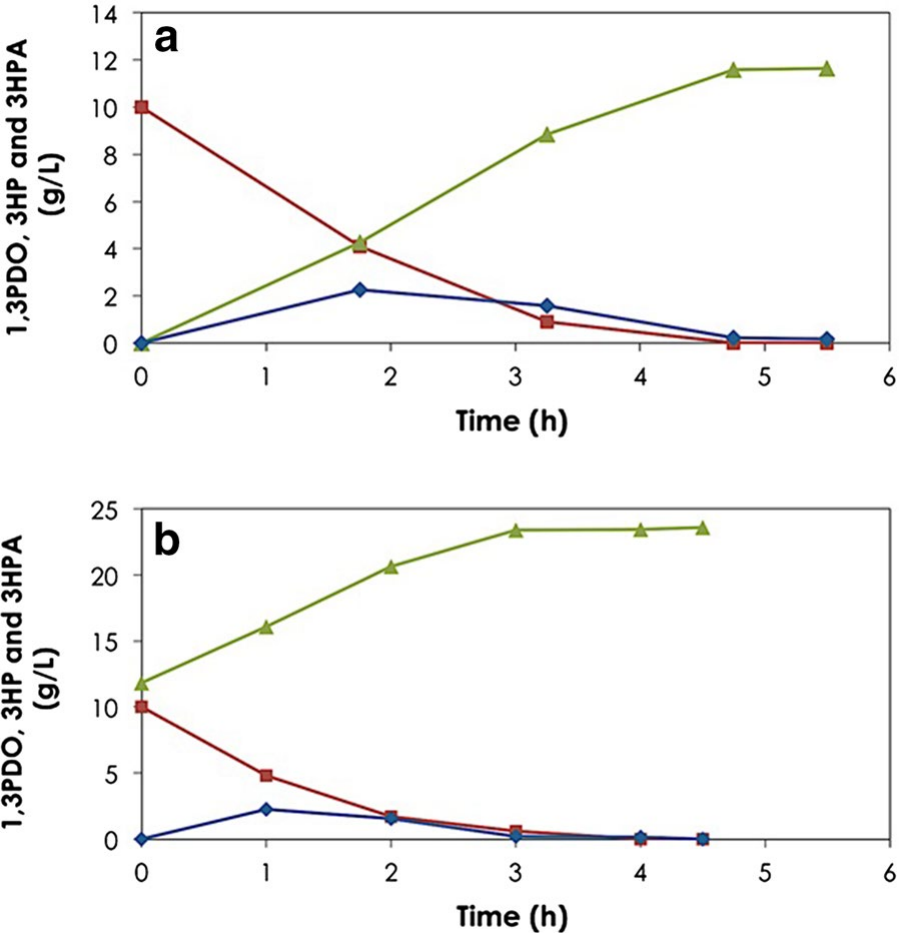
Biotransformation of 1,3PDO to 3HP. Batch biotransformation of 1,3PDO (*filled square*) to 3HP (*filled triangle*) via 3HPA (*filled diamond*) as intermediate using resting cells of *G. oxydans* (3.25 g_CDW_/L). **a** Model solution containing 10 g/L 1,3PDO and **b** 1,3PDO (10 g/L) in mixture with 3HP (11.8 g/L) obtained from glycerol biotransformation using resting cells of *L. reuteri*. The biotransformation was done in 3 L bioreactor with 1 L working volume. Temperature was controlled at 28 °C, stirrer speed at 800 rpm, airflow at 1 v/v/m, and pH at 5.5 through addition of 5 N NH_4_OH during the whole biotransformation process

Biotransformation of 1,3PDO performed under controlled aeration and pH in a bioreactor using 3.25 g_CDW_/L cells resulted in enhanced 1,3PDO oxidation and 3HP production rates (Fig. 6a). Consumption of the entire 1,3PDO (10 mg/mL) was achieved in 4.75 h at a volumetric rate exceeding 2.1 g/L h with formation of 3HP at a rate of 2.4 g/L h and a molar yield of ~1 mol_3HP_/mol_1,3PDO_. The specific consumption and production rates during the initial 3.25 h were 14.4 and 13.8 mg/g_CDW_.min, respectively.

Once the reaction kinetics for 1,3PDO was determined, the conversion of 1,3PDO present in a mixture with 3HP produced from biodiesel-derived glycerol by *L. reuteri* was investigated. No inhibitory effect of 3HP at an initial concentration of 11.8 g/L was observed on the reaction kinetics. The biotransformation of 1,3PDO in the mixture showed a similar profile to that of pure 1,3PDO. Quantitative conversion of 1,3PDO to 3HP was achieved yielding a final concentration of 23.6 g/L 3HP (Fig. 6b). The yield of 3HP with respect to the glycerol used reached approximately 1 mol/mol which is the highest reported so far (Additional file 1: Table S1), and the product had high purity exceeding 95 % as determined by HPLC. This solution was filtered and utilized as substrate for AA production.

### Catalytic dehydration of 3HP to AA

**Fig. 7:**
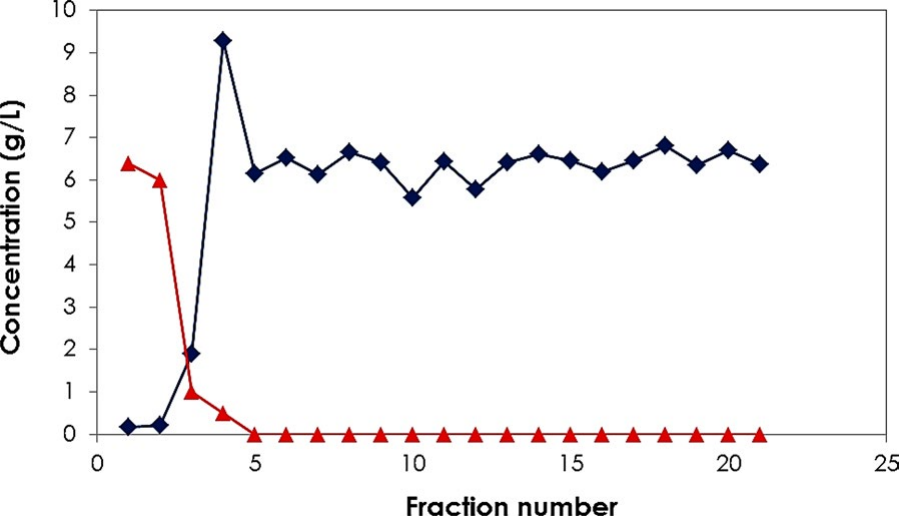
Catalytic dehydration of 3HP to AA. Continuous catalytic dehydration of 8 g/L 3HP (*filled traingle*) to AA (*filled diamond*) using a chromatographic column (300 × 7.8 mm) packed with 12 g TiO_2_ and placed in an oven at 230 °C. The 3HP solution was obtained as a product from glycerol via two-step biotransformation. Pre-heated 3HP solution (pH 5.7) was fed at a rate of 1.5 mL/h using a quantitative pump. The eluate containing AA was condensed in a cold water bath and collected in fractions

Dehydration for the introduction of double bond is one of the less investigated reactions through enzymatic catalysis. This could be a result of the high toxicity of these products such as acrolein, AA and methacrylic acid to the producing microorganisms [8]. On the other hand, chemical catalysis provides several green routes for production of these chemicals catalyzed by inorganic catalysts. Among the different processes for production of acrylic acid, the catalytic dehydration of 3HP over TiO_2_ gave the highest yield (Additional file 1: Table S2). Titanium dioxide (TiO_2_) is considered to be a green catalyst and is widely used in paints, cosmetics and food as whitening agent. We have reported earlier the catalytic dehydration of 3-hydroxy-2-methylpropionic acid over TiO_2_ at 210 °C to methacrylic acid with high purity exceeding 90 % and a yield over 85 % [31]. The same approach was for catalytic dehydration of 3HP to AA. The effect of the substrate flow rate on the dehydration step was tested in a continuous mode of operation. Table 2 summarizes the percentage of conversion and product yield in each case. Over 99.9 % 3HP conversion and 99.0 ± 5.1 % product formation was achieved using the substrate flow rate of 1.5 mL/h at 230 °C (Fig. 7).

**Table 2:** Effect of flow rate on dehydration of 3HP to AA using TiO_2_ at 230 °C

Run	Flow rate (mL/h)	Conversion (%)	Acrylic acid (mol %)
1	6	66.8 ± 2.3	25.4 ± 1.7
2	1.5	>99	99.0 ± 5.1

## Conclusions

**Fig. 8:**
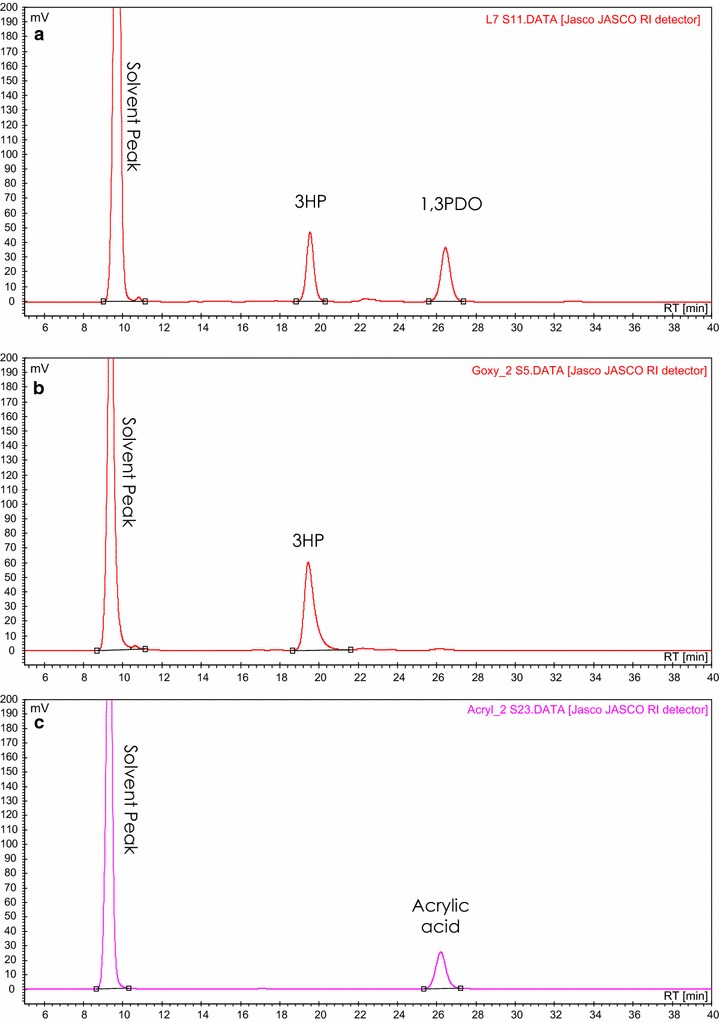
Chromatographic profiles for 1,3PDO, 3HP and AA production via the combined three-step process. **a** 3HP and 1,3PDO produced from glycerol using resting cells of *L. reuteri* DSM 20016. **b** 3HP produced from bioconversion of 1,3PDO in mixture with 3HP using resting cells of *G. oxydans*. **c** AA produced by catalytic dehydration of the resulting 3HP using TiO_2_ (3HP and AA peaks were confirmed using UV detection at 215 nm)

The present study provides a proof of concept for a novel green route for production of AA from biodiesel-derived glycerol via an integrated three-step process. The conversion of the substrate to the final product was mediated by seven enzymes in two whole cell biocatalysts and an inorganic catalyst. All the reactions were performed in aqueous medium, which resulted in high yields and purity of the final products without the need for further purification (Fig. 8). All the catalytic steps were performed at pH (5.5) in order to lower the base consumption and facilitate subsequent gas-phase dehydration step to AA [9].

According to a recent simulation study for AA production from glucose via 3HP using recombinant *E. coli*, the main concerns were the estimated high cost for downstream processing and the cost of nitrogen source required for microbial growth and maintaining the production [39]. For making the system reported in the present study economical and attractive for industrial scale production of 3HP and AA requires further investigations with respect to designing less expensive cultivation media for growing the microorganisms, and the bioconversion to handle higher substrate loads to obtain more concentrated product stream. This may also involve the use of engineered cells with overexpressed enzymes for improving the activity and productivity and lowering the product inhibition.

## Methods

### Materials

Glycerin tech (98 %), a co-product of biodiesel production process and 3-hydroxypropionic acid standard (30 % w/v) were provided by Perstorp AB, Sweden. Lactobacilli MRS broth (composition per liter: 10 g protease peptone, 10 g beef extract, 5 g yeast extract, 20 g dextrose, 1 g Tween 80, 2 g ammonium citrate, 5 g sodium acetate, 0.1 g magnesium sulfate, 0.05 g manganese sulfate and 2 g dipotassium phosphate) and Bacto yeast extract were products of Difco (BD laboratories, Detroit, Michigan, USA). 1,3-Propanediol, 28 % ammonium hydroxide, acrylic acid, TiO_2_ and glycerol (99 %) were obtained from Sigma–Aldrich (St. Louis, MO, USA) and glucose monohydrate from Prolabo (VWR International, Fontenay-sous-Bois, France), while 1,2-propanediol and phosphate salts were from Merck (NJ, USA).

### Microorganisms and culture conditions

Two microorganisms were used in this study, *L. reuteri* DSM 20016 and *G. oxydans* DSM 50049.

For preparation of *L. reuteri* pre-culture, MRS medium containing 1.5 g/L 1,2-propanediol was used. Stock culture of the microorganism in 20 % v/v glycerol (0.2 mL) was added to 20 mL of the culture medium in 30 mL serum bottles and incubated at 37 °C for 8 h. The resulting culture was used to inoculate another 20 mL medium (1 % v/v) and incubated under similar conditions for 8 h and then used as inoculum for biomass production step.

*G. oxydans* inoculum preparation and biomass production was done as described elsewhere [31], by transferring 1 mL stock culture in 20 % v/v glycerol to an agar slant, and after incubation at 28 °C for 2 days the culture on the agar surface was transferred to a 1 L baffled E-flask containing 100 mL culture medium (10 g/L yeast extract, 10 g/L glycerol and 5 g/L KH_2_PO_4_). The culture was placed in an incubator shaker (New Brunswick, Innova 4430, Edison, USA) at 200 rpm and 30 °C for 4 days and then used for biomass production step.

### Biomass production step

The production of *L. reuteri* and *G. oxydans* cells was done in a 3-L bioreactor (Applikon, Microbial Biobundle, The Netherlands). Monitoring and control of all the parameters was done through ez-control unit. Temperature was maintained via a heating blanket and a cooling finger, and pH was controlled using 5 N NH_4_OH.

For *L. reuteri*, 20 mL (1 % v/v) of the fresh pre-culture was aseptically added to 2 L fermentation medium containing 55 g/L MRS broth, 5 g/L 1,2-propanediol and glucose concentration was adjusted to 40 g/L. Stirrer speed was maintained at 200 rpm, temperature at 37 °C and pH at 5.5. Anaerobic conditions were maintained by continuous bubbling of nitrogen gas. Fermentation was continued for 10 h after which the broth was collected and centrifuged at 15,000×*g* for 10 min.

Production of *G. oxydans* cells was done as described elsewhere [31]. Seventy-five milliliters of the pre-culture was added to 1.5 L fermentation medium. The operational conditions used were temperature of 30 °C, pH 5.5, stirrer speed 500 rpm and airflow at 1 v/v/m during the entire cultivation. After cultivation for 2 days, cells were harvested as described earlier for *L. reuteri*.

### Fed-batch biotransformation of glycerol to 1,3PDO and 3HP using resting cells of *L. reuteri*

Biotransformation of glycerol was done in 3-L bioreactor (Applikon, The Netherlands) with 1 L initial working volume. The process was started by resuspending the harvested *L. reuteri* cells from the biomass production step in 1 L solution containing 2 g/L glycerol to a final density of 12 g_CDW_/L. After 3 h of batch biotransformation, glycerol (100 g/L) feed was started at a rate of 7.5 mL/h (0.75 g_Gly_/h) and was continued for 55 h. Samples were collected and analyzed for the concentrations of residual substrate and metabolites. The biotransformation conditions were the same (pH 5.5, 37 °C) as described in the *L. reuteri* biomass production step. The resulting solution containing equimolar concentrations of 1,3PDO and 3HP was collected, filtered through 0.2 µm filter and used as the substrate for resting cells of *G. oxydans*. The biotransformation kinetics were determined using the following equations:

- $$Production \, rate \, \left( {g/h} \right) = [\left( {P_{final} { \cdot }V_{final} } \right){-}\left( {P_{initial} { \cdot }V_{initial} } \right)]/\varDelta t$$
- $$Consumption \, rate \, \left( {g/h} \right) = [(S_{final} { \cdot }V_{final} ) - ((S_{feed} { \cdot }V_{feed} ) + \left( {S_{initial} { \cdot }V_{initial} } \right))]/\varDelta t$$
- $$Specific \, production \, rate, \, q_{P} \left( {mg/g_{CDW} { \cdot }h} \right) = production \, rate \, \left( {g/h} \right) \times 1000/x$$
- $$Specific \, production \, rate, \, q_{P} \left( {mg/g_{CDW} { \cdot }h} \right) = production \, rate \, \left( {g/h} \right) \times 1000/x$$
- $$Specific \, consumption \, rate, \, q_{S} \left( {mg/g_{CDW} \cdot h} \right) = consumption \, rate \, \left( {g/h} \right) \times 1000/x$$

where *P* and *S* are the concentrations of the products and substrate (g/L), respectively, *V* is the reaction volume, *x* is the amount of the biocatalyst (g_CDW_), and *∆t* is the time elapsed between the initial and final conditions (h).

### Batch biotransformation of 1,3PDO to 3HP using resting cells of *G. oxydans*

*G. oxydans* cells were obtained by centrifugation of the culture broth at 15,000×*g* for 2 min in Eppendorf tube. Oxidation of 1,3PDO (5–30 mg/mL) was studied using 2.6–6.5 mg (dry weight) resting *G. oxydans* cells per mL (1 mL reaction volume in 5 mL glass vials). The vials were placed in a thermomixer (Heidolph, Germany) at 28 °C and stirring at 800 rpm for maintaining aerobic conditions. Samples of 50 µL were collected and analyzed for concentrations of residual substrate and products.

Oxidative biotransformation of 1,3PDO to 3HP was also investigated using resting cells of *G. oxydans* with controlled pH and aeration. The biotransformation was done in a 3-L Applikon bioreactor with 1 L working volume under batch mode of operation. The experiment was started by resuspending 3.25 g_CDW_ of *G. oxydans* cells in 1 L solution containing 10.0 g/L 1,3PDO, and was maintained at 28 °C, pH 5.5 by addition of 5 N NH_4_OH, stirrer speed of 800 rpm and air flow at 1 L/min. The experiment was continued until 1,3PDO was entirely consumed.

In a similar manner, the conversion of 1,3PDO present in a mixture with 3HP produced from glycerol was performed. The *G. oxydans* cell pellet from the biomass production step was resuspended in the solution containing 10.0 g/L 1,3PDO and 11.8 g/L 3HP (equimolar) to a final density of 3.25 g_CDW_/L and biotransformation started under the same conditions as above.

Samples were collected and analyzed for the concentrations of 1,3PDO, 3HP and 3HPA. The initial reaction rates for the oxidation of 1,3PDO and 3HPA by ADH and ALDH were calculated for the initial 3 h of the reaction. For ADH, the reaction rate was calculated as the consumption rate of 1,3PDO, while for ALDH, the reaction rate was calculated as the production rate of 3HP. Since 3HPA is not available commercially, for plotting the relation between the substrate concentration (3HPA) and the initial reaction rate, the substrate concentration was calculated as the number of moles of 1,3PDO consumed after 3 h of biotransformation. Since 1,3PDO oxidation is a quantitative process ([3HPA]_initial_ = consumed [1,3PDO] = accumulated 3HPA + 3HP). The same strategy was used earlier for measuring the kinetics of the same enzymes [31].

The biotransformation kinetics were determined using the following equations:

- $$Volumetric \, production \, rate, \, Q_{p} \left( {g/L{ \cdot }h} \right) = [P_{final} {-}P_{initial} ]/\varDelta t$$
- $$Volumetric \, consumption \, rate, \, Q_{s} \left( {g/L{ \cdot }h} \right) = [S_{final} {-}S_{initial} ]/\varDelta t$$
- $$Specific \, production \, rate, \, q_{p} \left( {mg/g_{CDW} { \cdot }h} \right) = Q_{p} \times 1000/X$$
- $$Specific \, consumption \, rate, \, q_{s} \left( {mg/g_{CDW} { \cdot }h} \right) = Q_{s} \times 1000/X$$

where *X* is the cell density (g_CDW_/L).

### Catalytic dehydration of 3HP to AA

The 3HP solution obtained as a product from glycerol via two-step biotransformation was sterilized by filtration. A stainless steel tube (300 × 7.8 mm) was packed with 12 g TiO_2_ and placed in an oven. Pre-heated 3HP solution (8.00 mg/mL, pH 5.7) was fed into the pre-heated reaction tube using a quantitative pump (JASCO, Tokyo, Japan) at different flow rates for providing different residence times. The reaction temperature was 230 °C. The eluate containing AA was condensed in a cold water bath, collected in fractions and analyzed.

### Analyses and structural elucidation

#### Cell growth

Cell growth was followed by measuring optical density at 620 nm (OD_620_) using Ultrospec 1000 spectrophotometer (Pharmacia Biotech, Uppsala, Sweden) and then correlated with cell dry weight (CDW). The following equation describes the relation between the cell dry weight and optical density:

$$Cell \, dry \, weight \, \left( {g/L} \right) = OD \times 0.265$$

#### Measurement of substrates and products

Glycerol, 3HP, 1,3PDO and AA concentrations were determined by HPLC (JASCO, Tokyo, Japan) equipped with RI detector (ERC, Kawaguchi, Japan) and JASCO intelligent autosampler. Separation of the compounds was done using Aminex HPX-87H chromatographic column connected to a guard column (Biorad, Richmond, CA, USA). The column temperature was kept at 65 °C using chromatographic oven (Shimadzu, Tokyo, Japan). Samples were diluted with Millipore quality water and mixed with 10 % v/v sulfuric acid (20 µL/mL sample) and then filtered. Forty microliter sample was injected in 0.5 mM sulfuric acid mobile phase flowing at a rate of 0.4 mL/min.

For the determination of 3HPA concentration, the modified colorimetric method of Circle et al. 1945 [40] as described by Ulmer and Zeng (2007) [41] with acrolein as standard was used. Briefly, 200 µL of properly diluted sample was mixed with 150 µL of 10 mM DL-tryptophan solution in 50 mM HCl and 600 µL of concentrated HCl (fuming 37 %). The reaction mixture was incubated for 20 min at 37 °C. The produced purple color was then measured using spectrophotometer at 560 nm.

### Statistical analysis

The presented data are the average of two independent replicates ± standard deviation. Significant differences among treatment means were tested using the Student’s t test, with a level of significance of 0.05.

## Additional file

10.1186/s12934-015-0388-0 **Table S1.** Different processes for production of 3HP using wild-type and recombinant microorganisms. **Table S2.** Different chemical and biological processes for production of AA.
