# Supplementary material for: Bio-based 3-hydroxypropionic- and acrylic acid production from biodiesel glycerol via integrated microbial and chemical catalysis
Source: Microb Cell Fact. 2015 Dec 21;14:200. doi: 10.1186/s12934-015-0388-0 (PMC4687118; doi:10.1186/s12934-015-0388-0)
Supplement: Supplementary file 1 — 10.1186/s12934-015-0388-0 Table S1. Different processes for production of 3HP using wild-type and recombinant microorganisms. Table S2. Different chemical and biological processes for production of AA. [file 12934_2015_388_MOESM1_ESM.docx]

# Additional file 1:

# Bio-based 3-hydroxypropionic- and acrylic acid production from biodiesel glycerol via integrated microbial and chemical catalysis

Tarek Dishisha^1,2§^, Sang-Hyun Pyo^1^ and Rajni Hatti-Kaul^1^

^1^ Biotechnology, Center for Chemistry and Chemical Engineering, Lund University, SE-221 00 Lund, Sweden

^2^ Department of Microbiology and Immunology, Faculty of Pharmacy, Beni-Suef University, 62511 Beni-Suef, Egypt

^§^Corresponding author

Email addresses:

TD: [Tarek.Dishisha@biotek.lu.se](mailto:Tarek.Dishisha@biotek.lu.se)

SHP: [Sang-Hyun.Pyo@biotek.lu.se](mailto:Sang-Hyun.Pyo@biotek.lu.se)

RHK: [Rajni.Hatti-Kaul@biotek.lu.se](mailto:Rajni.Hatti-Kaul@biotek.lu.se)

## Additiional file 1: Table S1. Different processes for production of 3HP using wild-type and recombinant microorganisms

| **M.O.** | **C-Source** | **3HP** | | | | | | **Ref** |
| --- | --- | --- | --- | --- | --- | --- | --- | --- |
|  |  | **[3HP]** | ***Q_P_*** | ***Y*** | **Mode** | **Limitations** | **Req for AA***** |  |
| *rS. cerevisiae* | Glucose | 13.7 | 0.17 | 0.14 | Growing | Low *Y*, Low [3HP], Low *Q_P_*, low purity | DSP | [1] |
| *rE. coli* | Glu/Xyl | 29.4 | 0.54 | 0.36 | Growing | Low *Y*, low purity | DSP | [2] |
| *rE. coli* | Glycerol | 38.7 | 0.54 | 0.34 | Growing | Low *Y*, low purity | DSP | [3] |
| *rE. coli* | Glycerol | 31 | 0.43 | 0.34 | Growing | Low *Y*, low purity, low *Q_P_* | DSP | [4] |
| *rE. coli* | Glycerol | 16.3 | 0.34 | 0.28 | Growing | Low *Y*, low purity, low *Q_P_* | DSP | [5] |
| *rE. coli* | Glycerol | 40 | 1.26 | 0.26 | Growing | Low *Y*, low purity | DSP | [5] |
| *rE. coli* | Glycerol | 42.3 | 0.86 | 0.31 | Growing | Low *Y*, low purity | DSP | [6] |
| *rE. coli* | Glycerol | 57.3 | 1.59 | 0.86 | Growing | Low purity | DSP | [7] |
| *rE. coli* | Glycerol | 5 | 0.1 | 0.53 | Growing | Low *Y*, low purity, low *Q_P_*, Low [3HP] | DSP | [8] |
| *rE. coli* | Glycerol | 36.0 | 9 | 0.4 | Resting | Low *Y*, low purity | DSP | [9] |
| *rK. pneumonia* | Glycerol | 11.3 | 0.94 | 0.26 | Growing | Low *Y*, low purity, Low [3HP] | DSP | [10] |
| *rK. pneumonia* | Glycerol | 24.4 | 1 | 0.18 | Growing | Low *Y*, low purity | DSP | [11] |
| *rK. pneumonia* | Glycerol | 16 | 0.33 | 0.39 | Growing | Low *Y*, low purity, low *Q_P_* | DSP | [12] |
| *rK. pneumonia* | Glycerol | 28 | 0.58 | 0.39 | Growing | Low *Y*, low purity | DSP | [13] |
| *rP. denitrificans* | Glycerol | 4.9 | 0.25 | 0.66 | Growing | Low *Y*, Low [3HP], Low *Q_P_*, low purity | DSP | [14] |
| *wt L. reuteri* | Glycerol | 17 | 0.66 | 0.39 | Resting | Low *Y*, low purity | DSP | [15] |
| *rL. reuteri* | Glycerol | 20 | 1.08 | 0.39 | Resting | Low *Y*, low purity | DSP | [15] |
| *rL. reuteri* | Glycerol | 8.47 | 0.07 | 0.5 | Resting | Low *Y*, Low [3HP], Low *Q_P_*, low purity | DSP | [9] |
| *L. reuteri*  *rE. coli** | Glycerol | 1.1 | 0.06 | 0.68 | Resting | Low *Q_P_*, low [3HP] | -- | [16] |
| *wt L. reuteri*  *wt G. oxydans*** | Glycerol | 23 | 0.4 | 0.98 | Resting  Resting | Low *Q_P_* | -- | This study |
| * Three step process: Glycerol 🡪 3HPA 🡪 purification 🡪 3HP  ** Two step process: Glycerol 🡪 (3HP + 1,3PDO) 🡪 3HP  *** Prerequisite for acrylic acid production (DSP: Downstream processing) | | | | | | | | |

## Additional file 1: Table S2. Different chemical and biological processes for production of AA

| **Catalyst** | **Substrate** | **AA** | **Y** | **Ref** |
| --- | --- | --- | --- | --- |
| **Single step process from propene:** Polyvalent oxides with molybdenum oxide as the main catalyst and tellurium oxide as the promoter. | Propene 🡪 Acrolein 🡪 AA |  | 50-60% | [17] |
| **Two step gas-phase catalytic oxidation:**  **Step 1:** Air oxidation (CuO “Shell process”), (Bi_2_O_3_/MoO_3_ “Sohio process”) at 300 - 360°C and 1-2 atmospheric pressure  **Step 2:** Mo_12_V_1.9_Al_1.0_Cu_2.2_ at 300°C | Propene 🡪 acrolein 🡪 AA | 20 – 70% | 1^st^: 85% Acrolein+AA  2^nd^: 98% | [17, 18] |
| **High pressure Reppe Process)**  **-** Proceeds at 4 MPa and 235°C with a nickel oxide – copper(II) bromide. | Propene 🡪 Acetylene 🡪 AA | -- | 72% | [17, 19] |
| Two bed oxydehydration reaction:  1^st^ bed: tungstated zirconia type + silicon carbide at 300°C  2^nd^ bed: Mo_12_V_4.8_Sr_0.5_W_2.4_Cu_2.2_O_x_ in presence of O_2_ at 268°C | Glycerol | -- | overall: 75%  1^st^: 70%  2^nd^: 78.6% | [20, 21] |
| Acetoxylation of lactic acid to 2-acetoxypropionic acid using conc. sulfuric acid which upon pyrolysis yields acrylic acid | Lactate | -- | 1^st^: 90%  2^nd^: 95%  Overall: 92.5% | [22, 23] |
| Catalytic dehydration using calcium pyrophosphate catalyst at 375°C | Lactate | -- | 78% | [24] |
| Dehydration at 450°C and 100 MPa | Lactate | 32.5% | 13% | [25] |
| Catalytic dehydration over TiO_2_ at 180°C | 3HP | -- | 97.7% | [26] |
| - Fumaric acid was obtained from glucose using *rE. coli*  - Contacting fumaric acid with a sufficient amount of ethylene in the presence of a cross-metathesis transformation catalyst (Grubbs Ruthenium metathesis catalyst) at 1-5 atm , 0 – 50°C | Glucose 🡪Fumarate 🡪AA | -- | 1^st^: 6.4%  2^nd^: -- | [27] |
| 1^st^: Lactose 🡪 Propionate (PA): *L. bulgaricus + Propionibacterium shermani (coculture)*  2^nd^: Propionate 🡪 Acrylate: *Clostridium propionicum* | Lactose 🡪Lactate 🡪 (PA) 🡪 AA | 2.2 g/L | 1^st^ till PA: 8.9%  2^nd^: 18.5% | [28] |
| *rE. coli* | Glucose | 0.12 g/L | Low | [29] |
| *L. reuteri, G. oxydans,* TiO_2_ (at 230°C) | Glycerol | 6.2 g/L | 99% | This study |

**References:**

1. Borodina I, Kildegaard KR, Jensen NB, Blicher TH, Maury J, Sherstyk S, Schneider K, Lamosa P, Herrgård MJ, Rosenstand I, Öberg F, Forster J, Nielsen J: **Establishing a synthetic pathway for high-level production of 3-hydroxypropionic acid in *Saccharomyces cerevisiae* via β-alanine.** *Metab Eng* 2015, **27:**57-64.
2. Jung IY, Lee JW, Min WK, Park YC, Seo JH. **Simultaneous conversion of glucose and xylose to 3-hydroxypropionic acid in engineered *Escherichia coli* by modulation of sugar transport and glycerol synthesis.** *Bioresour Technol* 2015, **198:**709-716.
3. Rathnasingh C, Raj SM, Jo JE, Park S: **Development and evaluation of efficient recombinant *Escherichia coli* strains for the production of 3-hydroxypropionic acid from glycerol**. *Biotechnol Bioeng* 2009, **104:**729-739
4. Mohan Raj S, Rathnasingh C, Jung WC, Park S: **Effect of process parameters on 3-hydroxypropionic acid production from glycerol using a recombinant *Escherichia coli*.** *Appl Microbiol Biotechnol* 2009, **84:**649-657.
5. Jung WS, Kang JH, Chu HS, Choi IS, Cho KM: **Elevated production of 3-hydroxypropionic acid by metabolic engineering of the glycerol metabolism in *Escherichia coli*.** *Metab Eng* 2014, **23:**116-122.
6. Sankaranarayanan M, Ashok S, Park S: **Production of 3-hydroxypropionic acid from glycerol by acid tolerant *Escherichia coli*.** *J Ind Microbiol Biotechnol* 2014, **41:**1039-1050.
7. Kim K, Kim S-U, Park Y-C, Seo J-H: **Enhanced production of 3-hydroxypropionic acid from glycerol by the modulation of glycerol metabolism in recombinant *Escherichia coli*.** *Bioresour Technol* 2014, **156:**170-175.
8. Honjo H, Tsuruno K, Tatsuke T, Sato M, Hanai T: **Dual synthetic pathway for 3-hydroxypropionic acid production in engineered *Escherichia coli*.** *J Biosci Bioeng* 2015, **120:**199-204.
9. Yasuda S, Mukoyama M, Horikawa H, Toraya T, Morita H: **Process for producting 1,3-propanediol and or/3-hydroxypropionic acid.** US Patent 20070148749 A1; 2007.
10. Kumar V, Sankaranarayanan M, Jae KE, Durgapal M, Ashok S, Ko Y, Sarkar R, Park S: **Co-production of 3-hydroxypropionic acid and 1,3-propanediol from glycerol using resting cells of recombinant *Klebsiella pneumoniae* J2B strain overexpressing aldehyde dehydrogenase.** *Appl Microbiol Biotechnol* 2012, **96:**373-383.
11. Huang Y, Li Z, Shimizu K, Ye Q: **Simultaneous production of 3-hydroxypropionic acid and 1,3-propanediol from glycerol by a recombinant strain of *Klebsiella pneumoniae*.** *Bioresour Technol* 2012, **103:**351-359.
12. Ko Y, Ashok S, Zhou S, Kumar V, Park S: **Aldehyde dehydrogenase activity is important to the production of 3-hydroxypropionic acid from glycerol by recombinant Klebsiella pneumoniae.** *Process Biochem* 2012, **47:**1135–1143.
13. Ashok S, Sankaranarayanan M, Ko Y, Jae KE, Ainala SK, Kumar V, Park S: **Production of 3-hydroxypropionic acid from glycerol by recombinant *Klebsiella pneumoniae ΔdhaTΔyqhD* which can produce vitamin B_12_ naturally.** *Biotechnol Bioeng* 2013, **110:**511-524.
14. Zhou S, Catherine C, Rathnasingh C, Somasundar A, Park S: **Production of 3-hydroxypropionic acid from glycerol by recombinant *Pseudomonas denitrificans*.** *Biotechnol Bioeng* 2013, **110:**3177-3187.

# Dishisha T, Pereyra LP, Pyo SH, Britton RA, Hatti-Kaul R: Flux analysis of the *Lactobacillus reuteri* propanediol-utilization pathway for production of 3-hydroxypropionaldehyde, 3-hydroxypropionic acid and 1,3-propanediol from glycerol. *Microb Cell Fact* 2014, 13:1-10.

1. Sabet-Azad R, Sardari RRR, Linares-Pasten JA, Hatti-Kaul R: **Production of 3-hydroxypropionic acid from 3-hydroxypropionaldehyde by recombinant *Escherichia coli* co-expressing *Lactobacillus reuteri* propanediol utilization enzymes**. *Bioresour Technol* 2015, 180:214-221.
2. Ohara T, Sato T, Shimizu N, Prescher G, Schwind H, Weiberg O, Marten K, Greim H: **Acrylic acid and derivatives.** In *Ullmann's Encyclopedia of Industrial Chemistry.* Wiley-VCH Verlag GmbH & Co. KGaA: Weinheim; 2000.
3. Matar S, Hatch LF: *Chemistry of Petrochemical Processes.* Second edition: Gulf Professional Publishing; 2001.
4. Lin TJ, Meng X, Shi L: **Catalytic hydrocarboxylation of acetylene to acrylic acid using Ni_2_O_3_ and cupric bromide as combined catalysts.** *J Mol Catal A: Chem* 2015, **396:**77–83.
5. Dubois J-L: *Method for synthesis of acrolein from glycerol.* 2012. US Patent 8143454.
6. Contractor RM, Andersen MW, Campos D, Hecquet G, Pham C, Simon M, Stojanovic M, Kotwica R, Schirmann JP: *Vapor phase oxidation of acrolein to acrylic acid.* 2001. US patent 6310240.
7. Lilga MA, Werpy TA, Holladay JE. *Methods of forming alpha, beta-unsaturated acids and esters.* 2006. US Patent 6992209.
8. Fruchey OS, Malisezewski TA, Sawyer JE. *Acrylic acid from lactide.* 2013. WO/2013/036389.
9. Ghantani VC, Dongare MK, Umbarkar SB: **Nonstoichiometric calcium pyrophosphate: a highly efficient and selective catalyst for dehydration of lactic acid to acrylic acid.** *RSC Adv* 2014, **4:**33319-33326.
10. Aida TM, Ikarashi A, Saito Y, Watanabe M, Smith RLJ, Arai K: **Dehydration of lactic acid to acrylic acid in high temperature water at high pressures.** *J Supercrit Fluids* 2009, **50:**257–264
11. Lilga MA, White JF, Holladay JE, Zacher AH, Muzatko DS, Orth RJ: *Method for Conversion of ß-hydroxy Carbonyl Compounds.* 2010. US Patent 7687661.
12. Burk MJ, Pharkya P, van Dien SJ, Burgard AP, Schilling CH: *Methods for the synthesis of acrylic acid and derivatives from fumaric acid.* 2009. WO/2009/045637.
13. O'Brien DJ, Panzer CC, Eisele WP: **Biological production of acrylic acid from cheese whey by resting cells of *Clostridium propionicum*.** *Biotechnol Prog* 1990, **6:**237–242.
14. Chu HS, Ahn JH, Yun J, Choi IS, Nam TW, Cho KM: **Direct fermentation route for the production of acrylic acid.** *Metab Eng* 2015, **32:**23-29.
